# Percutaneous snare-retrieval of intracardiac thrombus under fluoroscopic and transesophageal echocardiography guidance: case report and systematic review

**DOI:** 10.3389/fcvm.2023.1127131

**Published:** 2023-05-09

**Authors:** Huaji Zhou, Bing Wang, Jun Pan, Chenyang Qiu, Xinyu Yu, Yangyan He, Qianqian Zhu, Lei Yu, Ziheng Wu, Donglin Li, Hongkun Zhang

**Affiliations:** ^1^ Department of Vascular Surgery, The NO.1 People’s Hospital of Pinghu, Jiaxing, China; ^2^ Department of Vascular Surgery, The First Affiliated Hospital, School of Medicine, Zhejiang University, Hangzhou, China

**Keywords:** intracardiac foreign body, transesophageal echocardiography, percutaneous retrieval, thrombus, snare retrieval

## Abstract

Intracardiac foreign bodies (IFB) are rare clinical conditions. There are now several reports on the percutaneous retrieval of IFB under fluoroscopy. However, some IFB are not radiopaque, and retrieval requires combined fluoroscopic and ultrasound guidance. We report the case of a bedridden 23-year-old male patient with T-lymphoblastic lymphoma treated with long-term chemotherapy. Ultrasound examination diagnosed a huge thrombus in the right atrium near the opening of the inferior vena cava which affected the patency of his PICC line. Ten days of anticoagulant therapy did not modify the thrombus size. Open heart surgery was not feasible because of the patient clinical condition. Snare-capture of the non-opaque thrombus was done from the femoral vein under fluoroscopic and ultrasound guidance with excellent outcomes. We also present a systematic review of IFB. We found out that percutaneous removal of IFBs is a safe and effective procedure. The youngest patient who received percutaneous IFB retrieval was 10 days old and weighed only 800 g, while the oldest patient was 70 years old. Port catheters (43.5%) and PICC lines (42.3%) were the most commonly found IFBs. Snare catheters and forceps were the most commonly used instruments.

## Introduction

Intracardiac foreign bodies (IFB) can result from invasive medical procedures or trauma (1, 2), and can cause infection, embolism, arrhythmia, and other complications (3, 4). Depending on their type, IFBs are diagnosed typically with x-ray, CT-scan, or ultrasound. The treatment is either conservative, surgical, or percutaneous removal (5, 6). In 1964, Thomas et al. reported the first percutaneous IFB retrieval without thoracotomy (7). Since then, there have been many case reports on the different modalities of IFB removals (8–11). Herein, we report an interesting case of percutaneous snare-retrieval of a non-radiopaque intracardiac thrombus under fluoroscopic and transesophageal echocardiography guidance and we present a comprehensive literature review.

## Case report

A severely ill 23-year-old male patient, diagnosed with T-lymphoblastic lymphoma, was receiving chemotherapy over his PICC line at our institution for 8 months.

**Figure 1:**
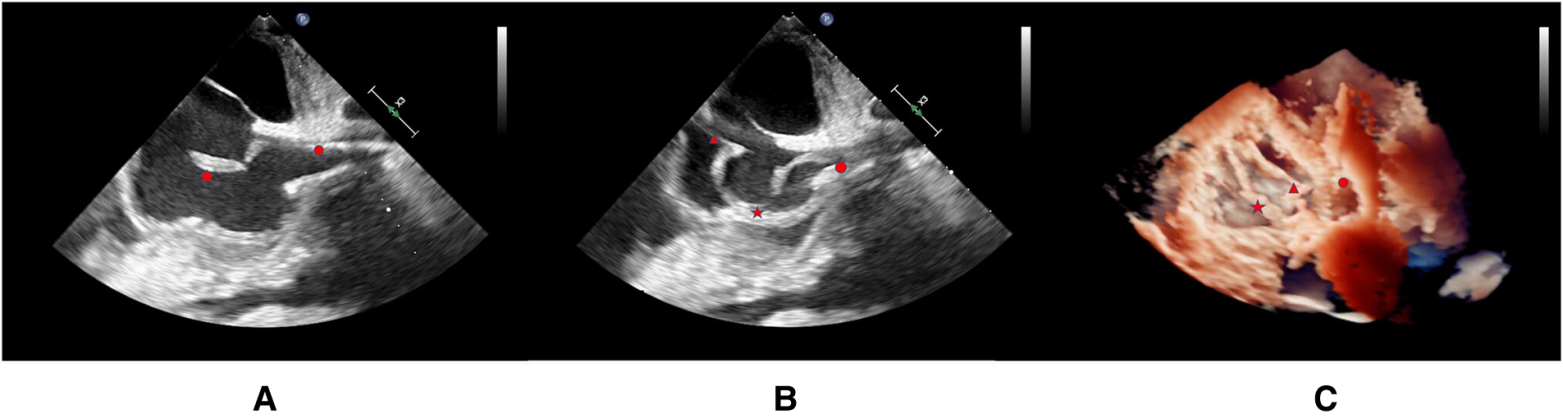
**The locations of the floating thrombus, gooseneck catcher, and PICC are indicated in the image by the five-pointed star, triangle, and circle, respectively. (**A**) Location of thrombus under transesophageal ultrasound (**B**) Location of thrombus and snare under transesophageal ultrasound (**C**) 3D reconstruction after endovascular treatment.

Transthoracic ultrasound was done for chest pain that cannot be relieved by thoracic puncture and drainage of a pleural. It showed a flocculent echo mass near the inferior vena cava in the right atrium. Transesophageal echocardiography (TOE) showed the thrombotic nature of the mass and described the IFB as a cord-shape formation in front of the caudal end of the PICC line with the tail floating into the right atrium, near the tricuspid valve (Figure 1). Anticoagulation therapy with low molecular weight heparin was started but no change in the size of the thrombus was observed after 10 days. A multidisciplinary team meeting was held to discuss the case, and it was decided that open-heart surgery is very risky for the patient. It was then decided to attempt a percutaneous retrieval under fluoroscopic and TOE guidance.

**Figure 2:**
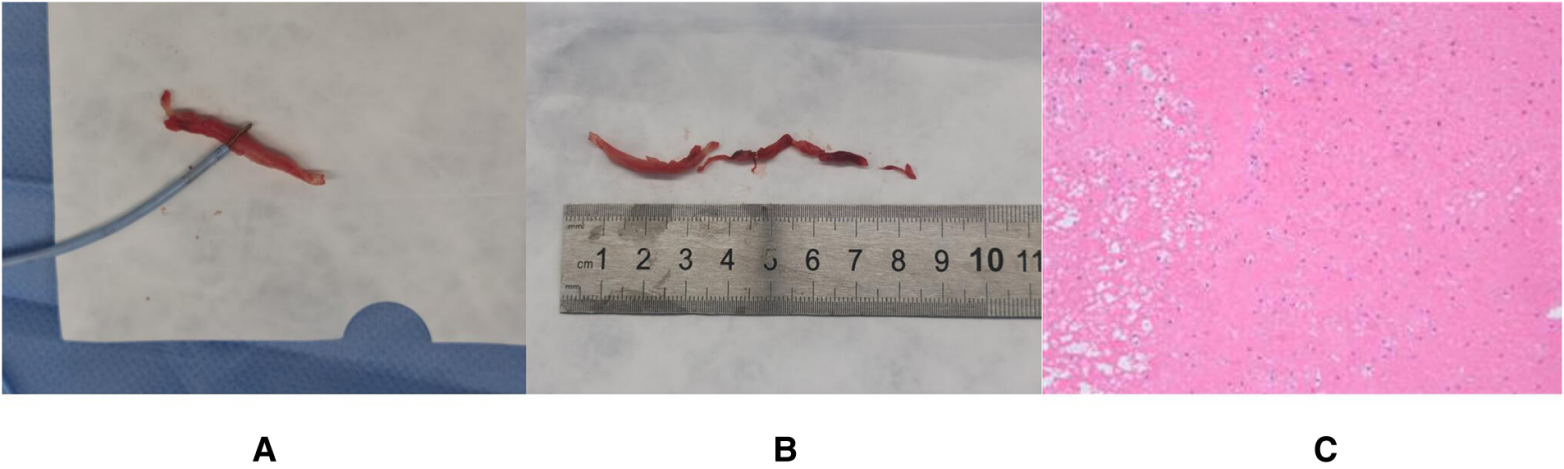
(**A**) Removed thrombus. (**B**) The size of the removed thrombus (**C**) Pathological findings of thrombus (mixed thrombus).

Under general anesthesia, the right femoral vein was accessed with a 10-Fr introducer (Terumo Corp.) and heparin was given. Baseline caval angiographies showed the PICC line in place but we were unable to delineate the location of the IFB. A Gooseneck snare catheter (LifeTech Scientific Corp.) was advanced to the right atrium (Supplementary Video S1). The snare and the thrombus were both localized on TOE. The operator performed repeated gentle grabs until the sonographer confirmed that the thrombus was captured (Supplementary Video S2). The thrombus was carefully exteriorized under fluoroscopic and TOE guidance. We did not identify a filling deficiency in the lumen of either pulmonary artery. TOE did not show residual thrombus at the caudal end of the PICC line and no thromboembolism occurred. Access hemostasis was obtained with manual compression. Anticoagulation therapy was maintained for 24 h. Postoperative pathology showed that the foreign body was old thrombus (Figure 2). The next day ultrasound showed PICC line patency and chemotherapy was restored. After 3 months, the patient had a bone marrow transplant and recovered well.

## Literature review

**Figure 3:**
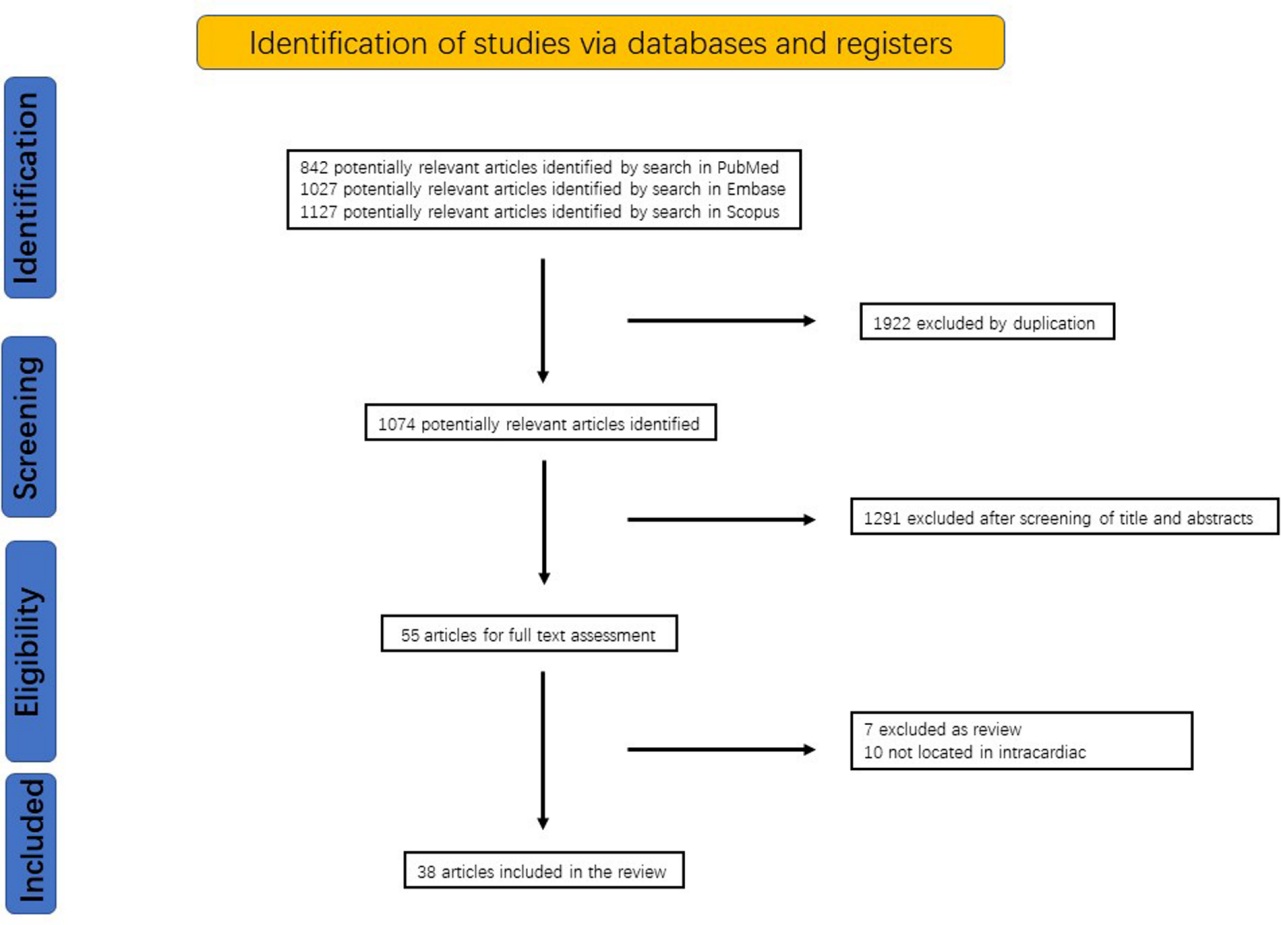
The preferred reporting items for systematic reviews flow diagram of studies on IFB.

We performed a systematic literature review in PubMed, Embase, and Scopus to identify English articles on endovascular treatment for IFBs from the inception of each database to the 8th of April 2023. Variations of the following search terms were used: percutaneous, retrieval, snare, intracardiac, etc. (Table 1). References of the selected articles was hand searched to look for more studies. Studies were selected for full-text review if their abstracts indicated that the IFB was intracardiac and treated percutaneously. Two independent investigators (B.W., and C.Q.) performed the study selection and data extraction. The search strategy identified 1,074 articles, of which 55 studies were selected for full-text review on the basis of the screening of title and abstract. Seventeen articles were excluded after the full-text review. In the end, a total of 38 reports (8, 9, 11–46) were published from 1975 to 2023 were selected and are comprehensively summarized in Table 2. The identification of studies was summarized in Figure 3.

**Table 1:** Search strategy.

**PubMed**
1. Foreign* [Title/Abstract] 2. Thromb* [Title/Abstract] 3. Clot* [Title/Abstract] 4. 1 OR 2 OR 3 5. Percutaneous* [Title/Abstract] 6. Endovasc* [Title/Abstract] 7. Snare* [Title/Abstract] 8. 5 OR 6 OR7 9. Retriev* [Title/Abstract] 10. Remov* [Title/Abstract] 11. 9 OR 10 12. Intracardiac* [Title/Abstract] 13. Heart* [Title/Abstract] 14. 12 OR 13 15. 4 AND 8 AND 111 AND 14
**Embase**
1. Foreign*:ab,ti 2. Thromb*:ab,ti 3. clot*:ab,ti 4. 1 OR 2 OR 3 5. Percutaneous*:ab,ti 6. Endovasc*:ab,ti 7. Snare*:ab,ti 8. 5 OR 6 OR 7 9. Retriev*:ab,ti 10. Remov*:ab,ti 11. 9 OR 10 12. Intracardiac* 13. Heart* 14. 12 OR 13 15. 4 AND 8 AND 11 AND 14
**Scopus**
1. TITLE-ABS-KEY ( foreign* ) 2. TITLE-ABS-KEY (thromb* ) 3. TITLE-ABS-KEY ( clot* ) 4. 1 OR 2 OR 3 5. TITLE-ABS-KEY ( percutaneous* ) 6. TITLE-ABS-KEY ( endovasc* ) 7. TITLE-ABS-KEY ( snare* ) 8. 5 OR 6 OR 7 9. TITLE-ABS-KEY ( retriev* ) 10. TITLE-ABS-KEY (remov*) 11. 9 OR 10 12. TITLE-ABS-KEY (intracardiac*) 13. TITLE-ABS-KEY (heart*) 14. 12 OR 13 15. 4 AND 8 AND 11 AND 14

**Table 2:** Detail information of included articles.

Author	Year	Sex	Age	Sample size	Symptom	Foreign body	Size	Location	Treatment	Retrieve	Complication
Fisher RG (26)	1975	Male	22	1	Asymptomatic	Central venous pressure catheter	/	RA + RV	Hook catheter and loop snare	Successful	None
Shaw TR (32)	1982	/	/	5	Asymptomatic	PICC	/	RA + RV	Forceps	Successful	None
Sproat IA (30)	1993	Male	49	1	Arrhythmia	Swan Ganz catheter	/	RV	Snare	Successful	None
Young T (44)	2003	/	10 days	1	NA	PICC	/	RA	Snare	Successful	None
Yen HJ (27)	2006	/	52	13	3/13 Local tenderness	PICC/Port/other	/	RA/RV/RA + RV	Loop snare	12/13 successfully retrieved	None
De Carolis MP (19)	2007	Female	27 weeks	1	NA	Umbilical arterial catheter	5 cm	RA	Goose Neck Snare	Successful	None
Bonvini RF (34)	2009	/	/	12	NA	Port	/	RA/RA + RV	Snare	11/12 successfully retrieved	1/12 arrhythmia
Grifka RG (33)	2013	Male	71	1	Chest pain	Methyl methacrylate glue fragment	60 mm	RA	Snare	Successful	None
Peng J (31)	2015	/	/	5	NA	PICC	/	RA + RV	Pigtail catheter	Successful	None
Baspinar O (35)	2015	/	/	2	NA	Port	/	RA	Snare	Successful	None
Mitsopoulos G (36)	2015	Female	47	1	Chest pain	Retained catheter	100 mm	RV	Snare	Successful	None
Ghaderian M (40)	2015	Female	12	1	Asymptomatic	Port	/	RA	Snare	Successful	None
Arindam Pande	2015	Female	55	1	Asymptomatic	PICC	/	RA + RV	Forceps	Successful	None
Bompotis G (28)	2016	Male	47	1	Asymptomatic	Tiny needle	11 mm	RV	Snare	Successful	Hemoptysis for a few days
Tajiri Y (39)	2016	/	52	13	1/13 Chest pain, 1/13 arrhythmia	Filter strut	/	LV	Snare + intracardiac echocardiography	Successful	None
Kalińczuk Ł (43)	2016	/	/	1	NA	Catheter fragment/port/guide-wire	/	RA/RV	Snare/pigtail/forceps	Successful	None
Figueredo	2016	Male	51	1	Arrhythmia	Thrombus		RA + RV	AngioVac aspiration system	Successful	None
Demirel A (18)	2016	Infant	28 weeks	1	Abdominal distension	Umbilical vein catheter	7 cm	RA	Cobra catheter and snare	Successful	None
Li PJ (20)	2016	Female	61	1	NA	PICC	15 cm	RA	Snare	Successful	None
Padala SK (9)	2017	Female	56	1	NA	Amplatzer septal occluder	26 mm	LA	Snare	Successful	None
Padiyath A (29)	2017	Male	6 weeks	1	NA	PICC	/	RA	Snare	Successful	None
Fanari Z (37)	2017	Male	63	1	NA	Closure device	/	LV	Forceps	Successful	None
Yoon SE (41)	2017	/	/	3	2/3 chest pain	Port	/	RA	Snare	Successful	None
Intagliata E (45)	2017	Female	56	1	Asymptomatic	Port	/	RA/RV	SNARE	Successful	None
Matthew N	2017	Male	69	1	NA	Thrombus	/	RZ	Suction device	Successful	None
Iglesias JF (47)	2019	Male	26	1	Pain and fever	Needle		RV	Conservative	Failed	/
Pazinato LV (25)	2020	/	/	14	1/14 Pleuritic pain and fever	Port/tunneled catheter/PICC	/	RA/RV	Snare/basket/pigtail	13/14 successfully retrieved	None
Sudhakar BGK (17)	2021	Female	60	1	Asymptomatic	PICC	/	RA	Snare	Successful	None
Haddad PG (14)	2021	Female	27	1	Asymptomatic	Stent	4 cm	RA	Snare and balloon	Successful	None
Shakerian B (24)	2022	Male	45	1	Asymptomatic	Hemodialysis catheter	23 cm	RA	Snare	Successful	None
Matsui Y (12)	2022	/	/	2	Asymptomatic	PICC	15 cm	RA	Long-shaft forceps	Successful	None
Trongtorsak A (13)	2022	Female	74	1	Chest pain and dyspnea	Bone cement material	/	RA	Gooseneck snare and ensnare	Successful	None
Sood S (42)	2022	/	17	1	Asymptomatic	PICC	/	RA + RV	Snare	Successful	None
Azeemuddin M (16)	2022	Female	67	1	Lethargy and asitia	PICC	/	RA	Snare	Successful	None
Bishnoi S (23)	2022	Male	32 weeks	1	Asymptomatic	PICC		RV	Gooseneck snare	Successful	None
Papatheodorou N (15)	2023	Male	58	1	Asymptomatic	PICC	/	RA + RV	Snare	Successful	None
Sasaki T (21)	2023	Female	75	1	Asymptomatic	PICC	/	RA	Snare	Successful	None
Luo H (22)	2023	Female	62	1	Precordial pain	stent	3 cm	RV	Goose neck snare	Successful	None

RA, right atrium; RV, right ventricle; NA, not applicable.

**Table 3:** Summary of included patients’ characters.

Item	*n*
Sample size	98
**Foreign body**	
Port catheter	42
PICC* (or fragment)	39
Thrombus	2
Needle	3
Septal occluder	2
Others	10
**Symptom**	
Asymptom	66
Chest pain	12
Arrhythmia	3
Others	17
**Treatment**	
Snare	84
Forceps	9
Pigtail	10
Suction device	2
**Outcome**	
Successful	93
Failed	5

PICC*, peripherally inserted central catheter.

We found out that the youngest patient who received percutaneous IFB retrieval was 10 days old and weighed only 800 g (44), while the oldest patient was 71 years old (33). Port catheters (42.9%) and PICC lines (39.8%) were the most commonly found IFB (34, 38, 46). There are also some reports of intracardiac needles and metal pieces due to drug use or trauma (47). In only 6 (5.1%) patients, percutaneous IFB removal was not successful (25, 27, 34, 43, 47). Snare catheters and forceps were the most commonly used instruments (25, 27, 46) Table 3.

## Discussion

IFBs can develop *in situ* or usually enter the heart via the circulatory system (48), most usually into the right chambers (49). IFBs can be asymptomatic (50), or they can cause infection, embolism, arrhythmia, and other issues (51, 52). In recent years, the increase of patients with chronic diseases requiring long-term use of drugs has led to more frequent use of long-term venous access systems (53, 54), with a subsequent increase in the incidence of IFB occurrence.

Nowadays, open thoracotomy, endovascular therapy, and conservative treatment are the 3 therapeutic alternatives for IFBs (5, 40, 55). Surgery is a more aggressive procedure, more expensive, and requires long recovery periods. On the other side, the endovascular procedure avoids certain major difficulties and has a claimed success rate of 87%–98% (7, 56, 57). Nowadays, endovascular approach is widely accepted as the first-line treatment for retrieving IFBs, while surgery is usually proposed as a second option (57–59). However, for certain foreign items (such as needles), the success rate cannot be guaranteed and is largely dependent on the doctor's experience and ability (48). There is no consensus regarding conservative treatment. It may rely on the patient's symptoms, the size of the foreign body, and the patient's willingness to undergo surgery.

Since 1964, several endovascular techniques and instruments have been developed and used to retrieve IFBs, including baskets, balloon catheters, grasping forceps, and tip-deflecting wires (7, 60, 61). According to the review, it is evident that the device most commonly used is a snare, which has been created for years by folding a guide wire of a tiny diameter into a loop. However, this stiff loop is vulnerable to damaging the vessel wall. A nondestructive soft metal ring that is welded to the distal end of a wallpaper wire is the modern trap (62). Balloon refers to the use of a flexible catheter with an expandable balloon at the head end. The angioplasty balloon catheter technique is used for cylindrical IFBs, with an inner lumen and a free edge (such as a stent) (57). The balloon is first inserted into the foreign body, and then inflated to form a grip with the foreign body. Baskets are devices that consists of two Nitinol wires looped together to form a basket shape (59). It is utilized to extract brief, tiny IFBs from smaller, tortuous, and distal vessels. Intravascular grasping forceps consist of two distally tapered metallic jaws (58). The proximal handle can be manually operated to adjust the bite force of the pliers. Nonetheless, this device must be operated by an experienced surgeon, as it is easy to cause vascular wall injury or perforation during the process of dragging foreign bodies (62).

X-rays and ultrasounds are typically used to detect IFBs. Fluoroscopy is typically used to perform percutaneous retrieval procedures because it makes it possible to see most metallic foreign bodies (56, 63, 64). However, there are additional reports of IFB retrieval paired with ultrasound guidance because the IFB is not radiopaque and it is challenging to pinpoint the IFB spatial location in the heart due to the heart's pounding (29, 30, 65). In this paper, the IFB was an ancient thrombus that had not responded to anticoagulant therapy and was not visible on fluoroscopy. Therefore, intraoperative TOE was helpful to locate the thrombus and guide the snare retrieval intervention.

Hematoma at the puncture site and intraoperative arrhythmias are the most prevalent consequences of percutaneous retrieval (66). However, such arrhythmias are typically self-limiting and may be connected with intraoperative instrument contact with heart-related tissues (67). From the review of the published reports, intravascular interventional therapy is safe and has a high success rate for retrieval IFBs. Its minimally invasive nature increases indications for even more vulnerable patients (68).

## Conclusion

Percutaneous retrieval of IFBs is a low-risk procedure. Intraoperative transesophageal echocardiography guidance can be helpful to determine the IFB spatial structure.
